# Perceptions and trends in the use of community pharmacies in Ghana

**DOI:** 10.1186/s40545-019-0186-x

**Published:** 2019-09-18

**Authors:** Grace Adjei Okai, Gordon Abekah-Nkrumah, Patrick Opoku Asuming

**Affiliations:** 1 Department Of Public Administration And Health Services Management, University Of Ghana Business School, P. O. Box 78, Legon, Accra, Ghana; 2 Department Of Finance, University Of Ghana Business School, P. O. Box 78, Legon, Accra, Ghana

**Keywords:** Community pharmacy, Perception, Community pharmacist, Minor ailments

## Abstract

**Objective:**

To examine the patterns in utilization of community pharmacies and perceptions of the general public towards community pharmacists’ role in health services delivery.

**Method:**

A cross-sectional household survey was conducted in Ga West district. A total of 497 adults (18 years and above) were chosen using a three-stage cluster random sampling technique. information on respondents’ contact with community pharmacies (i.e. 12 months prior to the study), reasons for visiting the pharmacies, factors influencing the choice of a particular pharmacy and perception towards community pharmacists’ roles were collected. Data collected were analyzed using stata version 14.

**Key findings:**

Out of the 497 respondents, 415 indicated that they had used pharmacies within the last 12 months prior to the study, while 82 indicated that they had not used the facilities within the same time frame. majority of the pharmacy users (33.7%) visited community pharmacies once a month. Approximately 84% of the pharmacy users frequently visited community pharmacies to get treatment for minor ailments. most users (about 75%) chose to visit a particular pharmacy as it was close to their home/workplace/hospital/clinic. More than half of the pharmacy users identified the pharmacist as the first point of contact in case of any drug-related problem. Less than half of the respondents (44.9%) perceived community pharmacists as health professionals with a good balance between health and business matters.

**Conclusion:**

The findings of the study suggest that beside the fact that majority of the respondents believe that community pharmacists are responsive, friendlier and have the capacity to handle minor ailments, they are indeed using community pharmacies for the treatment of minor ailments. It will therefore be important to develop appropriate policy and regulations that enables community pharmacies to adequately participate in the delivery of primary care and thereby improve population health.

## Introduction

Community pharmacies are an important aspect of the pharmaceutical sector and play a vital role in health care delivery. Also known as retail pharmacy, community pharmacy generally refers to a health care facility responsible for providing health services, which includes but not limited to the provision of drug information, clinical interventions, medication reviews, health screening, treatment of minor ailments, counselling on lifestyle modifications, provision of medicine and non-medicine treatments as well as documenting and preventing adverse drug reactions to the public [1–3]. Many of these facilities are located in various communities, have prolonged opening hours and do not require an appointment for consultation. Hence, they are considered more accessible than other health care facilities and are in a unique position to contribute immensely to health care delivery [4].

Community pharmacies are ideally positioned to provide health care advice to all category of people [5]. They also help to reduce costs associated with health care by lowering the occurrence of expensive forms of treatment such as hospital admissions and emergency room visits that occur due to inadequate drug use and adverse reactions [6]. Furthermore, community pharmacies have expedient hours of operation and provide services to clients with minimal waiting time [7]. Additionally, community pharmacies have a collective goal to enhance the well-being of the public and there is evidence to suggest that services provided in these facilities have resulted in improved patient care as well as optimal medical outcomes [8]. On the basis of availability, access, convenience and cost, some have suggested that using community pharmacies for the treatment of minor ailments can help optimize healthcare resources by reducing the demand for more costly health care options such as appointments with general practitioners [9–12].

Several studies conducted on community pharmacies show that they are the most frequently visited health facilities [13]. Purchases of prescription medicines and over-the-counter (otc) medicines in retail pharmacies have been found to be high among the reasons for pharmacy visits in several countries [14, 15]. Although the incidence of minor ailments is often high among a chosen population, use of retail pharmacies for advice and treatment of minor ailments, advice on medications and general health conditions have been found to be low in several developed and some developing countries [16–19]. Existing evidence suggest that majority of patients seeking care for minor ailment prefer to consult a general practitioner in a hospital, resulting in increased workload on practitioners, increased waiting time and consequently preventing those with potentially serious conditions from gaining access to appropriate care [20, 21].

Although the literature on community pharmacy utilization in developed countries is growing, evidence in Sub-Saharan Africa (SSA) is scanty [22, 23]. More importantly, there is currently no consensus on reasons for the use of community pharmacies, as the available evidence vary across countries. For example, evidence from Southern Africa suggests that, whereas in Zimbabwe, a few people preferred to utilize the pharmacy for advice and treatment of minor ailments, majority preferred to use community pharmacy for the same purpose in South Africa. Additionally, an essential component of community pharmacy is the role played by the pharmacist. For instance, the choice of a pharmacy is influenced by patient’s perception of the role of pharmacists [24]. Existing studies examining patient’s perception of the role of community pharmacists have mostly used a sample of patients or consumers within the premises of a pharmacy as well as those either entering or leaving the pharmacy premises [16, 17, 19, 25–27]. However, the use of consumers who are either entering or leaving the pharmacy premises creates possible sample selection and participant biases.

Although majority of pharmacies in Ghana are located in the Greater Accra and Ashanti regions [28], with more facilities springing up each year, there is relatively little information on how community pharmacy services are used by the general public. The only published study on community pharmacy we have identified in the existing literature, focused mainly on directions for community pharmacy service development, with little emphasis on utilization [5]. To fill the knowledge gap on utilization of community pharmacy in Ghana, the current study examines patterns in community pharmacy utilization and perceptions of the general public on the role of the community pharmacist in the delivery of health services using household data from the Ga West district of the Greater Accra region.

## Methods

Adults (18 years and above) residing in households in the Ga West district constitute the target population for the study. Children were excluded from the study due to issues of consent and also on the basis that they are unlikely to make decisions concerning their health. To obtain a representative sample, a three-stage cluster random sampling procedure was used. In the first stage, fifty (50) census enumeration area (EAs) or clusters were randomly sampled from the universe of EAs in the Ga West district, which according to the 2010 Ghana population and housing census, is 330 EAs [29]. The EAs were first stratified by type into three (type 1, 2 and 3) and probability proportionate to sample was used to sample the 50 EAs from the three types. After the selection of the EAs, a listing of all households in the sampled EAs was carried out to serve as the sampling frame for the second stage sample. In the second stage, 10 households were randomly sampled from each of the EAs. Finally, one household member aged eighteen (18) years and above in each of the selected households was randomly selected from the sample households and interviewed.

In all, 497 households were covered in the study. This is because in a particular EA, only 7 households were listed. This was because a large section of the EA had become a commercial area. In addition, residents of some of the selected households refused to participate in the study, whiles others were just not available. Ethical approval for the study was granted by the ethics committee of the humanities in the University of Ghana. Also, an approval letter was obtained from Ga West municipal assembly to conduct the study in the district. The aim of the study was explained to all the participants and a written consent form was completed by respondents before answering the questionnaire. It is also important to mention that using the 2010 population census figures for Ga West as a reference point (219,788), and the number of community pharmacies in Ga West as per data from the Pharmacy Council of Ghana, the Ga West district will have a community pharmacy to population ratio of one community pharmacy to 2817.79 population.

Prior to the data collection, 5 enumerators who are fluent in English, Twi, Dangme, Ewe and Ga (the predominant dialects in the study area) were trained for about six (6) hours on the procedures for data collection. The training focused on helping enumerators to understand (1) all facets of the questionnaire as well as how to ask each question and (2) rephrasing of certain questions during data collection and identifying local names for certain items that need translation in the local language. The data was collected using the computer-assisted personal interviews (CAPI) technology. The use of the CAPI meant that data collected by enumerators was received electronically on real-time and thereby eliminating potential errors and delays associated with manual collection of data. The questionnaire used for the study was adapted from previous literature sources [16, 19, 26, 30]. The questionnaire addressed the following issues: respondents contact with the pharmacy (i.e. 12 months prior to the study), reasons for visiting the pharmacy, factors influencing the choice of a particular pharmacy and perception of the community pharmacists’ roles in healthcare delivery, use of community pharmacy for minor ailments – suggested to be medical conditions that can be reasonably self-diagnosed and self-managed with over the counter medications [31]. The data collected was cleaned, checked for errors and analyzed using stata version 14.0. Frequencies, percentages of variables and other descriptive statistics were calculated. Besides the descriptive statistics, cross tabulation was used to examine associations between key socio-economic variables and use of community pharmacies.

## Results

### Socio-economic characteristics for respondents

The socio-economic characteristics of respondents are captured in Table 1. Out of the 497 respondents, 415 indicated that they had used pharmacies within the last 12 months prior to the study, Hence they were considered users of community pharmacy services. The remaining who had not used the services of any pharmacy within the same time frame were considered as non-users. majority of the respondents were christians (89%), 6.2% were muslim and 4% had no religion. most of the respondents (64.2%) were educated to the secondary level. A greater proportion (92.4%) of the respondents were employed and also, Most (74.7%) of the respondents lived in urban areas. A large number (57.5%) of the respondents were enrolled on a health insurance scheme. Equally, a large number of the total respondents (70.4%) pointed out that they had suffered some form of minor ailments 12 months prior to the study. The mean age of the respondents (see Table 2) was 40.9 years, with the mean distances to the nearest pharmacy being 0.5 kilometers for users and 1 kilometer for non-users of pharmacy services.

**Table 1:** 1 Socio-economic characteristics of respondents

	Users		Non-Users		Overall		*P*-value
	Frequency	%	Frequency	%	Frequency	%	
Gender							0.071^*^
Male	163	39.3	41	50	204	41	
Female	252	60.7	41	50	293	59	
Marital Status							0.104
Married	238	57.4	43	52.4	281	56.5	
Consensual Union	8	1.9	2	2.4	10	2	
Divorced	20	4.8	2	2.4	22	4.4	
Separated	37	8.9	2	2.4	39	7.8	
Widowed	33	8	9	11	42	8.5	
Never Married	79	19	24	29.3	103	20.7	
Religion						0.521	0.521
No Religion	18	4.3	2	2.4	20	4.02	
Christian	370	89.2	74	90.2	444	89.3	
Muslim	26	6.3	5	6.1	31	6.2	
Traditional	1	0.2	1	1.2	2	0.4	
Educational Level							0.536
None	43	10.4	12	14.6	55	11.1	
Primary	63	15.2	15	18.3	78	15.7	
Secondary	270	65.1	49	59.8	319	64.2	
Tertiary	39	9.4	6	7.3	45	9.1	
Employment Status							0.214
Unemployed	29	7	9	11	38	7.7	
Employed	386	93	73	89	459	92.3	
Location Type							0.153
Rural	105	25.3	25	30.5	130	26.2	
Urban	310	74.7	57	69.5	367	73.8	
Insurance Status							0.001^**^
Yes	253	61	33	40.2	286	57.5	
No	162	39	49	59.8	211	42.5	
Type Of Insurance							0,718
Private Insurance	1	0.4	0	0	1	0.3	
NHIS	252	99.6	33	100	285	99.7	
Had Minor Ailments							0.000^**^
Yes	343	82.7	7	8.5	350	70.4	
No	72	17.3	75	91.5	147	29.6	

Source: Constructed by authors based on field data ^*^ Significant At 1% ^**^ Significant At 10%. (*N* = 497)

**Table 2:** Descriptive statistics of respondents

	Users	Non-users	Overall
Variable	Mean SD	Mean SD	Mean SD
Age of respondent (years)	40.8 13.2	41.2 12.8	40.5 13.1
Distance to pharmacy (Km)	0.5 2.5	1.0 1.9	0.6 2.4

Source: Constructed by authors based on field data

Beside the overall frequency in Table 1, a disaggregation of the results between users and non-users reveal some interesting findings. For example, 82% of the respondents who used a community pharmacy in the last 12 months, were people who had minor ailments. additionally, 61% of respondents who used community pharmacies in the last 12 months had some form of insurance, With 0.4 and 99.6% being holders of private and national health insurance scheme -nhis (i.e. a publicly operated social health insurance scheme) policies respectively. It is important to emphasise that both the private insurance and the nhis cover pharmaceutical cost. Also 75% respondents interviewed lived in urban areas, 93% of them were employed, a little over 80% of them had either primary or secondary education, and finally about 61% of them were females.

### Community pharmacy utilization

#### Reasons for not using community pharmacy

For respondents who indicated that they had not used community pharmacy 12 months prior to the study, majority (22.3%) indicated distance as the reason for not using the services of a pharmacy. This was followed by no pharmacy in the community (17%), lack of money (15.2%) and other reasons (16.1%). Lack of trust in pharmacists/personnel in pharmacy and religious beliefs constituted 13.4 and 8.9% respectively (see Table 3).

**Table 3:** Reasons for not using community pharmacy (*N* = 82)

Reasons	Frequency	Percentage
Lack of trust in pharmacists/personnel In the pharmacy	15	13.4
Pharmacy is far	25	22.3
There is none in this community	19	17
I don’t have money	17	15.2
I prefer herbal medicine	5	4.4
I don’t like medicine	3	2.7
Religious beliefs	10	8.9
Other	18	16.1
Multiple responses, so the cumulative percentage will be more than 100%		

Source: Constructed by authors based on field data

#### Reasons for using Community pharmacy

Majority of the pharmacy users (about 84%) stated that they frequently visit community pharmacies to get treatment for minor ailments, 55% for the purchase of prescription medicines, 48% for the purchase OTC products, 10.8% for advice and treatment for another person and 9.4% for advice on general health conditions. only 1.9 and 1% reported visiting the pharmacy primarily to get home diagnostic devices and to purchase para-pharmaceutical products respectively.

#### Common conditions people seek treatment for in community pharmacies

Tables 4, 5 shows that the most common conditions respondents sought treatment for in pharmacies were body aches and pains representing 84.8%, followed by colds (43.9%), coughs (31.8%) and stomach pain (29.4%). Diarrhea, heart burns, sore throats, skin diseases and other ailments accounted for 3.1, 2.9, 1.7, 1.2 and 3.1% respectively.

**Table 4:** Most common reasons for visiting community pharmacies

Reasons	Frequency	Percentage
To get advice and treatment for minor ailments	348	83.9
To purchase prescribed medications	228	54.9
To get medications that i can purchase without prescription (OTC products)	199	48.0
To seek for advice or treatment for another person	45	10.8
Get advice on general health condition	39	9.4
Get advice on management and use of medications	19	4.6
Get home diagnostic devices (Glucometer Etc.)	8	1.9
Obtain para-pharmaceutical products (eg. Sunscreens, Cosmetics, Baby Care products)	4	1.0
Multiple responses, so the cumulative percentage will be more than 100%		

Source: Constructed by authors based on field data

**Table 5:** Conditions people seek treatment for in community pharmacies

Health conditions	Frequency	Percentage
Coughs	132	31.8
Colds	182	43.9
Sore throats	7	1.7
Body aches and pains	352	84.8
Heartburns	12	2.9
Stomach pain	122	29.4
Diarrhoea	13	3.1
Skin diseases	5	1.2
Other	13	3.1
Multiple responses, so the cumulative percentage will be more than 100%		

Source: Constructed By Authors Based On Field Data

#### Factors influencing the choice of a particular pharmacy

Most pharmacy users (approximately 75%) chose to visit a particular pharmacy as it is close to their home, workplace, hospital or clinic, followed by good relationship with pharmacist/staff (32.5%), good and competitive prices (27.5%) and good range of products and services available (17.3%). other reasons for patronizing a particular pharmacy were the pharmacist’s ability to answer any drug-related question (16.9%), quick services (12.3%), attractive pharmacy appearance (1.4%) and other reasons (0.2%). Table 6 presents descriptive statistics of factors influencing the choice of a particular pharmacy.

**Table 6:** Factors that make one choose a particular pharmacy to visit

Factors	Frequency	Percentage
Location (close to work, home, hospital or clinic)	326	75.2
Good and competitive prices	114	27.5
Quick services	51	12.3
Good range of products and services available	72	17.3
Good relationship pharmacist and/staff	135	32.5
Attractive pharmacy appearance	6	1.4
Knowledge of the pharmacist and his/her ability to answer any drug/disease related question	70	16.9
Other	1	0.2
Multiple responses, so the cumulative percentage will be more than 100%		

Source: Constructed by authors based on field data

#### Respondents perceptions of pharmacy conditions

As per the results in Table 7, majority of the respondents (45.8%) who used a pharmacy in the last 12 months indicated that waiting time in the pharmacy is short, with 22.4 and 30.4% suggesting that waiting time in the pharmacy is very short and normal respectively. Very few respondents (1.4%) rated the waiting time as long. The average waiting time in the pharmacy for respondents (not reported) was 6.9 minutes. In addition to waiting time, majority of the respondents who had used a pharmacy in the last 12 months (73.3%) indicated that lack of privacy in the pharmacy will not deter them from using the facility for treatment of minor ailments, with the remaining indicating otherwise. this suggest that majority of pharmacy users were really not concerned about privacy. Most importantly, 81.4% of the respondents (pharmacy users) indicated that pharmacy staff were friendlier than hospital staff whilst the remaining thought otherwise.

**Table 7:** Respondence perception of pharmacy conditions

Variables	Frequency (*N* = 415)	Percentage
Perception of waiting time		
Very short	93	22.4
Short	190	45.8
Normal	126	30.4
Long	6	1.4
Privacy in the pharmacy		
Concerned about privacy	110	26.5
Unconcerned about privacy	305	73.5
Friendliness of pharmacy staff		
Yes	338	81.4
No	77	27.6

Source: Constructed by authors based on field data

#### Public Perception of the role of Community Pharmacists

The results (not reported in the table below) suggest that 59.3% of those using pharmacy services see the pharmacist as the first person to contact for any drug-related question. This was followed by a physician (32%), other persons (4.6%), family and friends (3.1%) and nurse (1%). As per the results in Table 8, majority of the respondents (75%, representing strongly agree and agree) considered community pharmacists to have the expertise to diagnose and treat minor ailments with 11.6 and 13.5% being neutral and disagreeing respectively. Additionally, majority of the respondents (58.5%, representing strongly agree and agree) perceived that community pharmacists have the knowledge to provide advice on general health conditions. Also, less than half of the respondents (48.7%, representing strongly agree and agree) perceived community pharmacists as health professionals with a good balance between health and business matters (see Table 8).

**Table 8:** Perceptions concerning community pharmacists roles

Perceptions	Responses	Users		Non-Users		Overall	
		Freq.	%	Freq.	%	Freq.	%
Pharmacists’ have the expertise to diagnose and provide treatment for minor ailments	Strongly agree	14	3.4	4	4.9	18	3.6
	Agree	297	71.6	39	47.6	336	67.6
	Neutral	48	11.6	23	28	71	14.3
	Disagree	55	13.3	16	19.5	71	14.3
	Strongly Disagree	1	0.2	0	0	1	0.2
Pharmacists’ have the knowledge to provide advice on general health conditions	Strongly agree	8	1.9	0	0	8	1.6
	Agree	235	56.6	40	48.8	275	55.3
	Neutral	120	28.9	27	32.9	147	29.6
	Disagree	51	12.3	15	18.3	66	13.3
	Strongly Disagree	1	0.2	0	0	1	0.2
Pharmacists’ have a good balance between health matters and making money	Strongly agree	2	0.5	0	0	2	0.4
	Agree	200	48.2	21	25.6	221	44.5
	Neutral	199	48	55	67.1	254	51.1
	Disagree	14	3.4	5	6.1	19	3.8
	Strongly Disagree	0	0	1	1.2	1	0.2
Pharmacists’ know a lot about drugs and are concerned about and committed to caring for the public	Strongly agree	6	1.4	0	0	6	1.2
	Agree	191	46	20	24.4	211	42.5
	Neutral	198	47.7	56	68.3	254	51.1
	Disagree	20	4.8	6	7.3	26	5.2
	Strongly Disagree	0	0	0	0	0	0
Pharmacists’ are more concerned with the health of patients than making money	Strongly agree	6	1.4	2	2.4	8	1.6
	Agree	173	41.7	16	19.5	189	38
	Neutral	210	50.6	57	69.5	267	53.7
	Disagree	26	6.3	7	8.5	33	6.6
	Strongly Disagree	0	0	0	0	0	0

Source: Constructed by authors based on field data

## Discussion

The findings of the study suggest that majority of the respondents use the services of community pharmacies and that distance and other availability proxies (availability of pharmacies in the community, money and trust in the pharmacist) constitute key factors that influence the use of pharmacy services in general and choice of a particular pharmacy. At the disaggregated level, the results suggest that among pharmacy users, those who have health insurance, live in an urban area, are educated (i.e. primary and secondary education) or employed are more likely to use the services of pharmacies. The results also suggest that majority of the respondents went to the community pharmacy on issues of minor ailment in addition to the purchase of prescription or over the counter medications. Most importantly, majority of the respondents believe that the pharmacist should be the first person to call, on issues related to medicines and that they are knowledgeable enough to provide advice on general health conditions.

As per the results, socio-economic characteristics such as urban residence, being employed, having insurance and either primary or secondary education influences the decision to use pharmacy services. In the case of urban residence, a major reason may be due to the fact that majority of pharmacies (80%) are in the two biggest cities (Accra and Kumasi), in Ghana, thus giving urban residents better access to pharmacies [32]. Additionally, being employed, having health insurance as well as having either primary or secondary education is correlated with household wealth and therefore the possibility of removing access constraints. Hence, the positive influence of these socio-economic variables on the use of community pharmacies. This finding is in line with the existing health service utilization literature, which has substantial evidence to the fact that the above socio-economic variables are correlated with utilization of health services [33–35].

Also, the use of pharmacy by majority of the respondents for the treatment of minor ailments is not unexpected. In many developing countries such as Ghana, poverty constrain a lot of sick people from being able to access the services of hospitals. Besides, congestion in hospitals and poor quality of service make hospitals a place of last resort especially for those with minor ailment. It is therefore not surprising that majority of the users of community pharmacy services go there to seek treatment for minor ailments. This however, vary from existing findings from Bosnia, Malta and Qatar [17, 30, 36], where the purchase of prescription and OTC medicines constitute the predominant reasons for visiting pharmacies. The variation in findings suggests that the level of use of community pharmacies is determined by the extent to which such pharmacies act as substitute for main stream hospitals. In Ghana, where the stock of health facilities are limited, coupled with access bottlenecks, users are more likely to consider pharmacies to be good substitutes for mainstream hospitals as in the current case.

The findings also suggest that access is crucial to the use of community pharmacies for the different reasons for which they are used. The findings suggest that distance and nearness to either home/school/workplace constitute a key driver of the use of community pharmacies. As already indicated, there is substantial evidence in the health service utilization literature in Ghana [33–35] that suggest that availability and accessibility to health services influences utilization. Beside Ghana, evidence from Malta, West Bank-Palestine and Kuwait [16, 19, 26] suggest that the location of a pharmacy is key in determining utilization.

It is important to emphasise that the perception of short waiting times at the pharmacy is crucial to the usage and development of community pharmacies. In both developed and developing countries, longer waiting time constitute a key challenge for accessing health services [7, 20, 21]. It is normal to find long ques in out-patient departments in several hospitals in Ghana. Thus, the realization that community pharmacies tend to have shorter waiting times could be important to improving access to community pharmacies in particular and thereby improve access to healthcare in general. Additionally, the finding that majority of community pharmacy users in the study sample see community pharmacist to be friendly is very positive. There is substantial evidence in the healthcare quality literature to suggest that users’ perception of healthcare quality and consequently perception of service quality is influenced by the attitudes and behavior (courtesy, friendliness etc.) of health professionals. Thus, the friendly attitudes of personnel in community pharmacies, could be crucial in improving the levels of utilization of community pharmacies [37].

Surprisingly, users of community pharmacies do not seem to be concerned about issues of privacy. On face value, one may argue that this may probably be good, in that poor privacy conditions may not adversely affect utilisation. However, the insensitivity of users to violations of privacy by operators of community pharmacies may mean that rogue operators may take advantage of such insensitivity on the part of users to the detriment of the general public. It will therefore be important that the regulator implement interventions and monitor such interventions to ensure that the privacy of users of community pharmacies are respected. Indeed, infringements on privacy, if not checked by the regulator, can get out of hand and can adversely affect the use of community pharmacies by the general public.

Also worthy of note is the fact that respondents in the study have so much trust in the community pharmacist that they are willing to contact him first, on drug-related issues as well as using the pharmacy for advice and treatment of minor ailments. Respondents’ perception that pharmacists have the knowledge to provide advice on general health conditions as well as the expertise to provide advice on management and use of medications is equally important. The positive perception may be due to respondents’ trust in the pharmacists’ ability to answer their questions and also provide appropriate counselling. This may also be related to the issue of accessibility and availability, which is problematic in developing countries like Ghana. Thus, it is not uncommon for patients to choose pharmacies both as a first point of call for medicine-related issues and treatment of minor ailments. The current findings however vary from evidence from Kuwait [16], United Kingdom [38], and Lebanon [25], but consistent with evidence from Iraq (Ibrahim et al.).

The finding that less than half of the respondents believe that a pharmacist is a health professional whose main interest is caring for the public rather than making money could have adverse implications for the utilisation of community pharmacies if not corrtected. This is particularly important in a country like Ghana, where prices of medicines are very high (estimated to be about 50% more) compared to other West African countries [39]. The higher prices which also reflect higher profit margins, especially at the retail level (i.e. where most community pharmacies operate), may be responsible for the negative opinion respondents have about the commitment of pharmcists to the delivery of healthcare.

A comparison of the current results to evidence in the eixting literature suggest that country level charateristics play a key role in the nature and extent of use of community pharmacies. For example, the current results which is consistent with evidence from other countries countries (e.g. West Bank Palestine and Iraq) suggest that the use of community pharmacies is high in countries with poorer and weaker health system. Thus, in a developing country like Ghana, with relatively weaker health systems, improving the delivery and regulation of care within the community pharmacy ecosystem could be crucial in expanding availbility and access, improving equity, and consequently population health.

## Conclusion

The study examined patterns of utilization of community pharmacies and the perceptions of the general public on the role of pharmacist in health service delivery. The results of the study suggest that majority of the respondents use community pharmacies for medicines-related advice and treatment of minor ailments, with availability and accessibility being key drivers influencing the use of community pharmacies. Additionally, the findings of the study suggest that a minority of the respondents see pharmacist as professionals who are mainly interested in healthcare delivery as opposed to making money. These findings are important for policy development, especially in the area of improving availability and access to primary healthcare services. As earlier suggested, community pharmacies may constitute an important alternative to main stream hospitals, such that the level of congestion in main stream hospitals could be reduced. Thus, knowledge of the current findings can be seen as a window of opportunity that can be relied upon by policy-makers to evolve appropriate policy that may help bring community pharmacies into the delivery of specific primary healthcare services. The findings equally have implications for regulation in the community pharmacy space. For example, the idea of using community pharmacies to handle minor ailments is essential but could results in serious consequences if the regulatory environment is not strong enough. Additionally, the fact that users don’t seem to see privacy arrangements as crucial in a community pharmacy may intrinsically reduce the motivation of community pharmacies to self-regulate. This may also mean the need to strengthen the regulatory capacity of existing regulatory institutions in the health sector to ensure that relevant and appropriate standards are enforced within the community pharmacy space.

## Data Availability

The data for the study can be released upon reasonable request to the corresponding author.
